# Identifying Frail Populations for Disease Risk Prediction and Intervention Planning in the Covid-19 Era: A Focus on Social Isolation and Vulnerability

**DOI:** 10.3389/fpsyt.2021.626682

**Published:** 2021-08-20

**Authors:** Chiara Cerami, Marco Canevelli, Gaia Chiara Santi, Caterina Galandra, Alessandra Dodich, Stefano F. Cappa, Tomaso Vecchi, Chiara Crespi

**Affiliations:** ^1^Scuola Universitaria Superiore IUSS Pavia, Pavia, Italy; ^2^Cognitive Computational Neuroscience Center, IRCCS Mondino Foundation, Pavia, Italy; ^3^Department of Human Neuroscience, Sapienza University of Rome, Rome, Italy; ^4^National Center for Disease Prevention and Health Promotion, Italian National Institute of Health, Rome, Italy; ^5^Neurogenetic Research Center, IRCCS Mondino Foundation, Pavia, Italy; ^6^Center for Neurocognitive Rehabilitation - CIMeC, University of Trento, Rovereto, Italy; ^7^Dementia Research Center, IRCCS Mondino Foundation, Pavia, Italy; ^8^Department of Brain and Behavioral Sciences, University of Pavia, Pavia, Italy; ^9^Cognitive Psychology Center, IRCCS Mondino Foundation, Pavia, Italy

**Keywords:** COVID-19, frailty, social vulnerability, psychosocial variables, social isolation

## Abstract

The early identification of fragile populations in the Covid-19 era would help governments to allocate resources and plan strategies to contain consequences of the pandemic. Beyond frailty, social vulnerability to environmental stressors, such as the social distancing enforced to reduce the SARS-CoV2 contagion, can modify long-term disease risk and induce health status changes in the general population. We assessed frailty and social vulnerability indices in 1,258 Italian residents during the first lockdown phase *via* an on-line survey. We compared indices taking into account age categories and gender. While frailty showed a linear increase with age and was greater in females than in males, social vulnerability was higher in young adults and elders compared to middle aged and older adults, and in males than females. Both frailty and social vulnerability contributed in explaining the individual perception of the impact of Covid-19 emergency on health, which was further influenced by proactive attitudes/behaviors and social isolation. Social isolation and loneliness following the Covid-19 outbreak may exert dramatic psychosocial effects in the general population. The early detection of vulnerable categories, at risk to become ill and develop long-lasting health status changes, would help to prevent consequences on general well-being by allocating resources to targeted interventions managing psychosocial distress and increasing young adults and elderly resilience toward the post-Covid-19 crisis.

## Introduction

After more than a year from the discovery of the first infected cases in China, the new coronavirus (SARS-CoV-2) is continuing to claim victims all around the world. Beyond the world health emergency, the coronavirus disease 19 (Covid-19) pandemic is bringing down the global economy and threatening the stability of social systems. As the outbreak increased in each country, forced measures of social distancing and isolation were progressively adopted by national governments. In Italy, the abrupt wave of infected cases recorded in Northern regions on February 2020 imposed extreme containment measures and social distancing for 3 months. All Italian resident inhabitants were bordered within their houses. The massive lockdown disposed by the Italian government (DCPM #iorestocasa—I stay at home—March 9, 2020) forced thus millions of people to change work habits, daily routines, and lifestyles. This large-scale catastrophic event occurring within a very short amount of time hit thousands of singles and families and dramatically decreased the psychosocial well-being of the population.

The rapid diffusion of the SARS-CoV-2 contagion among the population required to reallocate available healthcare resources (e.g., intensive care unit and emergency dedicated personnel) and plan strategies to sustain difficult medical and ethical choices (e.g., guidance for the use of mechanical ventilation, triage systems). In this perspective, yet in the first phases of the pandemic the screening of frailty has been suggested as possible key tool to assist clinicians in decision-making (https://www.nice.org.uk/guidance/ng159/resources/covid19-rapid-guideline-critical-care-in-adults-pdf-66141848681413, accessed on 27 March 2020).

People above 65 years of age actually represent the population with higher risk of poor outcomes in case of SARS-CoV-2 contagion (1). They account for more than 80% of Covid-19 related deaths (2). However, the characterization of risk profiles and the provision of care in the case of older people cannot be properly based on mono-dimensional criteria (e.g., chronological age) that are poorly informative of the overall health status and needs of the aging individual. According to a recent meta-analysis on clinical characteristics of Covid-19 patients, age, male gender, hypertension and diabetes are significantly associated with increased mortality (3). In this regard, the adoption of the frailty construct may allow to target choices and interventions to the clinical and biological complexity of the individual, in the premise of a person-centered approach (4).

Frailty is a condition characterized by reduced homeostatic reserves and increased vulnerability to stressors exposing the individual to negative outcomes (5). It is a widely used clinical measure, both in geriatrics and other medical specialties, resulting from combining different age-related biological determinants leading to decreased functional reserve capacities (6). It is growingly recognized as a valid proxy of the individual risk profile toward adverse health outcomes (e.g., disability and/or mortality) (7). Frailty measures have become crucial instruments for planning and delivering services and are recommended by scientific society guidelines to quick and reliably screen populations for clinical vulnerability and to orient the triage procedures (https://www.nice.org.uk/guidance/ng191, published on 23 March 2021, updated on 8 April 2021). The Clinical Frailty Scale (CFS) has proven to provide more informative data than single measures of cognition, function or comorbidity in assessing medium-term risk of death for Covid-19 infection [(8); NICE https://www.nice.org.uk/guidance/ng159/resources/covid19-rapid-guideline-critical-care-in-adults-pdf-66141848681413 published on 20 March 2020, updated on 12 February 2021]. Moreover, the use of a Frailty Index at patient hospital admission during the first weeks of Covid-19 pandemic in Italy helped the clinical decision-making process, predicting mortality and ICU admission (9). However, the individual's risk of negative health-related outcomes is not influenced only by his/her biological asset and complexity. A significant role is also played by diverse social circumstances and psychological determinants that are not captured by biologically-oriented definitions of frailty (10).

Frailty and social vulnerability can be both summarized by using a deficit accumulation approach, i.e., arithmetically counting the biological and psychosocial negative attributes presented by the individual. Based on this model, the more deficits an individual has the more he/she will vulnerable to stressors and at risk for unfavorable outcomes (11). Previous studies have shown that the indexes resulting from this approach (i.e., frailty index and social vulnerability index) predict a range of health outcomes (12–14). A person-centered approach assessing different dimensions that may influence health status—considered as biological, psychological and social well-being—should therefore be promoted in order to assess more thoroughly the risk of short- and long-term adversities and the outcome of pharmacological and non-pharmacological interventions.

In the present study, we evaluated how frailty and social vulnerability, both operationally defined using a cumulative approach, influence the psycho-socio-emotional dimensions and the individual perception of Covid-19 impact on health. Moreover, we assessed the impact of age and sex on these two constructs. We predicted that, in line with current literature, frailty would increase with age and females would result frailer than males (15–17). We expected that, in the light of the social distancing imposed during the lockdown phase, social categories whose significant relationships were held outside the family (e.g., young adults) or away from their living context (e.g., elders) would result vulnerable compared to people who have active and strong social ties within their home (e.g., middle aged and older adults living with their children). Moreover, we hypothesized that the frailty and social vulnerability may interact with the psychological and emotional asset of the individual in influencing his/her perception of the pandemic.

## Participants and Methods

### Participants

Immediately after the lockdown phase disposed by Italian government on March 9, 2020 (DCPM #iorestoacasa—I stay at home), we launched the PsyCOVID study (https://wprn.org/item/428452), aiming at evaluating changes in habits, routines and psychosocial dimensions in the Italian population during the social distancing period [see baseline findings at Cerami et al. (18)].

As we reported earlier (18), we conducted an anonymous on-line survey among Italian residents Between March 14 and 31, 2020. We used convenience sampling, selecting participants based on their accessibility and proximity to the research group. We created the survey using Google Forms and distributed it through a freely accessible link (https://forms.gle/5f3yH3aTNJYEuJ7B9). We distributed the survey link *via* written invitations through e-mails, WhatsApp and social networks. We then asked initial participants to diffuse the questionnaire through their social networks. Eligibility criteria were age (18 years of age or older) and place of residence (Italy). The PsyCovid Study was approved by the IUSS-University of Pavia Ethics Committee and performed in accordance with relevant guidelines/regulations. All study participants provided their informed consent to the experimental procedure and they did not receive any incentive to take part in the study.

The response rate was 98% and was calculated as the ratio of the number of complete responses to the total number of potential participants who had the chance to access the first page of the survey (18). Non-responders were persons who did not provide their informed consent to participate or who declared an age <18 years old.

A total of 1,258 adult Italian residents completed the survey (71.5% females; mean age: 43 ± 13.5; age range: 18–81). Table 1 provides details about the socio-demographic characteristics of the sample.

**Table 1:** Sample description.

**Characteristics**	**No. (and %) of respondents**
**Sex**	
Male	359 (28.5)
Female	899 (71.5)
**Age**	
Young adults (18–34 y)	405 (32.2)
Middle adults (35–49 y)	472 (37.5)
Old adults (50-64 y)	269 (21.4)
Elders (>65 y)	112 (8.9)
**Education**	
Elementary school (5 y)	2 (0.2)
Secondary school (8 y)	30 (2.4)
High school (13 y)	352 (28.0)
Graduate school (16–18 y)	580 (46.1)
Postgraduate school (>18 y)	293 (23.3)
**Occupation**	
Student	88 (7.0)
**Housemaker**	33 (2.6)
Unemployed	53 (4.2)
Employee	576 (45.8)
Manager	105 (8.3)
Freelance	230 (18.3)
Professor or researcher	37 (2.9)
Retired	117 (9.3)
**Job field**	
Industry	114 (9.1)
Financial and economy	118 (9.4)
Communication industry	60 (4.8)
Art and manufacturing	59 (4.7)
**Humanities**	199 (15.8)
Non-profit	99 (7.9)
Construction	25 (2.0)
Trade	65 (5.2)
Healthcare	185 (14.7)
Education and university	61 (4.8)
Public services	62 (4.9)
Others	205 (16.3)
**Geographic area (place of residence)**	
Northern Italy	832 (66.1)
Center Italy	115 (9.1)
Southern Italy	311 (24.7)

*The table reports demographic features of the sample (n = 1,258)*.

### Measures

#### Frailty

Frailty was measured by computing a Frailty Index (FI) following a standard procedure (19, 20). A total of 30 variables, representing symptoms, clinical signs, comorbidities, and impaired functions, were considered. For each item, we assigned a score 0 in the absence and 1 in the presence of the deficit. The FI score was then calculated by dividing the sum of the deficits presented by each participant by the total number of variables measured (i.e., 30). The variables incorporated in the FI are listed in Appendix A in Supplementary Material.

#### Social Vulnerability

Social vulnerability was operationalized analogously to frailty, by calculating a cumulative social vulnerability index (SVI). Thirty self-reported variables pertaining to social and psychological factors were considered. Each item was scored as 0 (absent) or 1 (present). The total number of deficits presented by the subject was then divided by the total number of deficits considered, yielding a continuous SVI score ranging from 0 to 1. The variables incorporated in the SVI are reported in Appendix B in Supplementary Material.

Of note, the FI and SVI were mutually exclusive, with no deficit overlap between the two instruments.

#### Psycho-Socio-Emotional Dimensions

In addition to data on socio-demographic characteristics, the questionnaire recorded information about different psycho-socio-emotional dimensions, relevant for emergency settings and post-traumatic situations (i.e., loneliness, empathic skills, coping strategies, alexithymia) (21–25). To collect information about these psycho-socio-emotional dimensions we used a battery of validated questionnaires in Italian language. Loneliness was assessed with the Italian Loneliness Scale (ILS) (26), including the three sub-scales (General Loneliness, Emotional Loneliness, Social Support). We used the Empathic Concern (EC) and Perspective Taking (PT) sub-scales of the Interpersonal Reactivity Index (IRI) (27) to capture emotional and cognitive facets of empathic abilities, respectively. Coping strategies were investigated with the short version of the Coping Orientation to the Problems Experienced (COPE-NVI-25) scale (28), measuring different coping behaviors or styles toward problems and stressful events, reflected in 5 sub-scores (Positive attitude, Problem orientation, Transcendence orientation, Social support, Avoidance strategies). Finally, we recorded information about individuals' ability to identify and describe emotions experienced by one's self or others with the Toronto Alexithymia Scale (TAS-20) (29).

#### Perceived Impact of Covid-19 Outbreak on Health

We assessed the perceived impact of Covid-19 outbreak on health with a 4-item scale (*average interitem covariance* = 0.34; *Cronbach's alpha or* α = *0*. *74*) [see (18)]. This scale required participants to rate the perceived severity of Covid-19 outbreak for health at the local (item 1: city or town), regional (item 2), and global (item 3: national; item 4: international) levels, on a 5-point Likert scale (0 = not serious at all; 4 = extremely serious). The individual global score results by summing up the item ratings (range 0–16).

### Statistical Analysis

We performed statistical analyses using SPSS (https://www.spss.it/) and STATA (https://www.stata.com/).

Since a small percentage of data were missing in any analysis (<2% of cases), we dropped cases with missing values *via* list-wise deletion. We set statistical significance at *p* < 0.05 for all statistical tests. We calculated descriptive statistics including frequencies and percentages for categorical variables, and mean and standard deviation for pseudo-continuous variables. We estimated interindividual differences in FI and SVI with a two-way MANOVA, considering sex and age categories (young adults: 18–34 y.o., middle aged adults: 35–49 y.o., old adults: 50–64 y.o., elders: >64 y.o.) as fixed factors.

Then, in order to reduce dataset complexity and optimize interpretability of results, we applied a Principal Component Analysis (PCA) on variables reflecting psycho-socio-emotional dimensions. In particular, after assessing the suitability of the correlation matrix (Keiser-Meyer-Olkin Measure of Sampling Adequacy = 0.702; Bartlett's test of sphericity <0.001), we performed a PCA on the scores of 11 variables, including the three ILS sub-scores, EC and PT sub-scores from the IRI, the five COPE-NVI-25 sub-scores and TAS-20 global score. Both the scree plot and the Kaiser-Guttman criterion (i.e., components with eigenvalue >1) converged in determining the number of components to be retained (=3). We used an orthogonal rotation (Varimax) to facilitate the interpretation of the resulting components (30). Loading factors of the three components obtained were then used in the subsequent correlation and mediation analyses. Correlation analysis (Pearson's r coefficient) was carried out to evaluate the relationship linking FI and SVI with PCA components (C1: *Proactivity*; C2: *Isolation*; C3: *Inactivity*) and the perceived impact of Covid-19 outbreak on health.

Finally, in the light of the correlation results, we assessed two different mediation paths *via* the sgmediation package in STATA. The first (*Social Vulnerability Model*) tested the indirect effect of C1 (*Proactivity*) on the relationship between SVI and perceived impact of Covid-19 outbreak on health. The second mediation path (*Frailty Model*) assessed the indirect effect of C2 (*Isolation*) on the relationship linking FI and perceived impact of Covid-19 outbreak on health.

## Results

### Sample Distribution of FI and SVI

**Figure 1:**
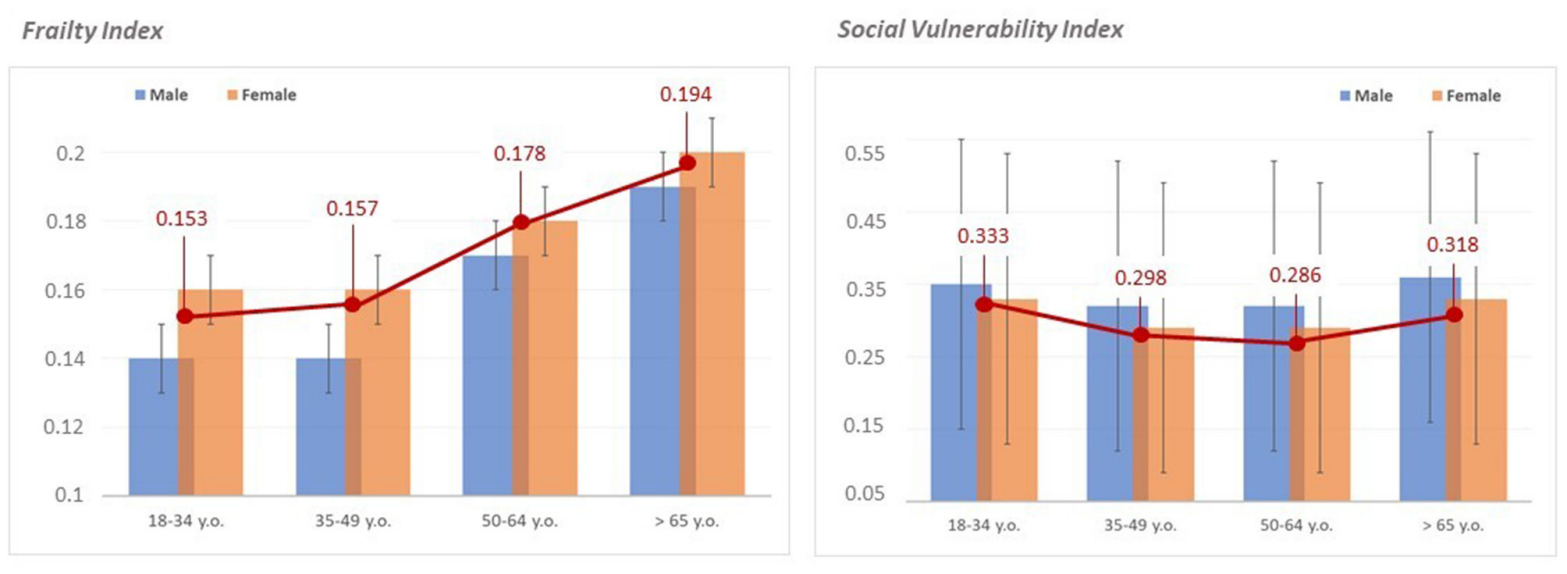
Distribution of frailty and social vulnerability by age categories and gender. The figure illustrates the mean distribution of the frailty index (on the left) and the social vulnerability index (on the right) in the whole sample (red lines) grouped by age categories (18–34 y.o. = young adults; 35–49 y.o. = middle adults; 50–64 y.o. = old adults; >65 y.o. = elders). Blue and orange histograms indicate distribution patterns of frailty and social vulnerability indices in males and females, respectively.

Descriptive statistics are illustrated in Table 2 and Figure 1.

**Table 2:** Descriptive statistics of frailty and social vulnerability indices.

		**Frailty index**						**Social vulnerability index**					
		**Males**			**Females**			**Males**			**Females**		
		**Mean**	**SD**	**Range**	**Mean**	**SD**	**Range**	**Mean**	**SD**	**Range**	**Mean**	**SD**	**Range**
Age	18–34	0.14	0.07	0.03–0.37	0.16	0.08	0.03–0.43	0.35	0.1	0.10–0.60	0.33	0.1	0.07–0.70
	35–49	0.14	0.07	0.03–0.37	0.16	0.1	0.03–0.83	0.32	0.11	0.03–0.60	0.29	0.1	0.03–0.60
	50–64	0.17	0.09	0.03–0.40	0.18	0.11	0.00–0.63	0.32	0.12	0.03–0.63	0.29	0.11	0.03–0.67
	>65	0.19	0.1	0.03–0.37	0.2	0.11	0.07–0.53	0.36	0.11	0.17–0.57	0.33	0.12	0.13–0.67

*The table reports mean, standard deviation (SD) and score range of both indices in the sample (n = 1,258), grouped by age categories and gender*.

### Age and Gender Effects on FI and SVI

The two-way MANOVA showed a significant multivariate effect of gender [∧ = 0.984; *F*_(2, 1249)_ = 10.036, *p* < 0.001] and age categories [∧ = 0.957; *F*_(6, 2498)_ = 9.253, *p* < 0.001] on both FI and SVI. However, the interaction between gender and age categories was not significant [∧ = 0.999; *F*_(6, 2498)_ = 0.127, *p* = 0.993]. Univariate results confirmed gender and age categories effects on both FI [*sex: F*_(1, 1250)_ = 5.951, *p* = 0.015, with a greater FI in females than males; *age categories*: *F*_(3, 1250)_ = 9.211, *p* < 0.001], and SVI [*sex: F*_(1, 1250)_ = 14.040, *p* < 0.001, with a greater SVI in males than females; *age categories*: *F*_(3, 1250)_ = 9.309, *p* < 0.001]. *Post-hoc* analysis (Bonferroni *post-hoc* test) of univariate results taking into account between-group differences in age categories showed that while FI was significantly higher in old adults (50–64 y.o.) and elders (>64 y.o.) compared to young (18–34 y.o.) and middle aged adults (35–49 y.o.) (Figure 1), SVI was significantly higher in young adults and elders compared to middle aged and old adults (Figure 1).

### Relationship Between FI and SVI, Psycho-Socio-Emotional Variables, and Perceived Impact of Covid-19 on Health

The PCA reduced the original 11 psycho-socio-emotional variables into 3 non-collinear components, explaining the 61% of the overall variance (Supplementary Table 1). Component 1 (C1: *Proactivity*) included variables related to empathy, social support, active and positive coping strategies, denoting an internal locus of control. Component 2 (C2: *Isolation*) included two loneliness variables. Finally, Component 3 (C3: *Inactivity*) encompassed variables related to alexithymia, transcendent or avoidance coping stiles, indicating an external locus of control.

Correlation analyses assessing the relationship between FI, SVI, the three psycho-socio-emotional components (C1, C2, C3) and the perceived impact of Covid-19 on health are reported in Supplementary Table 2. In particular, we observed that both FI (*p* < 0.05) and SVI (*p* < 0.01) were significantly correlated with the perceived impact of Covid-19 on health. Based on the correlation patterns emerged, we selected a definite set of variables and tested two mediation paths in one model, with the perceived impact of Covid-19 outbreak on health as dependent variable.

**Figure 2:**
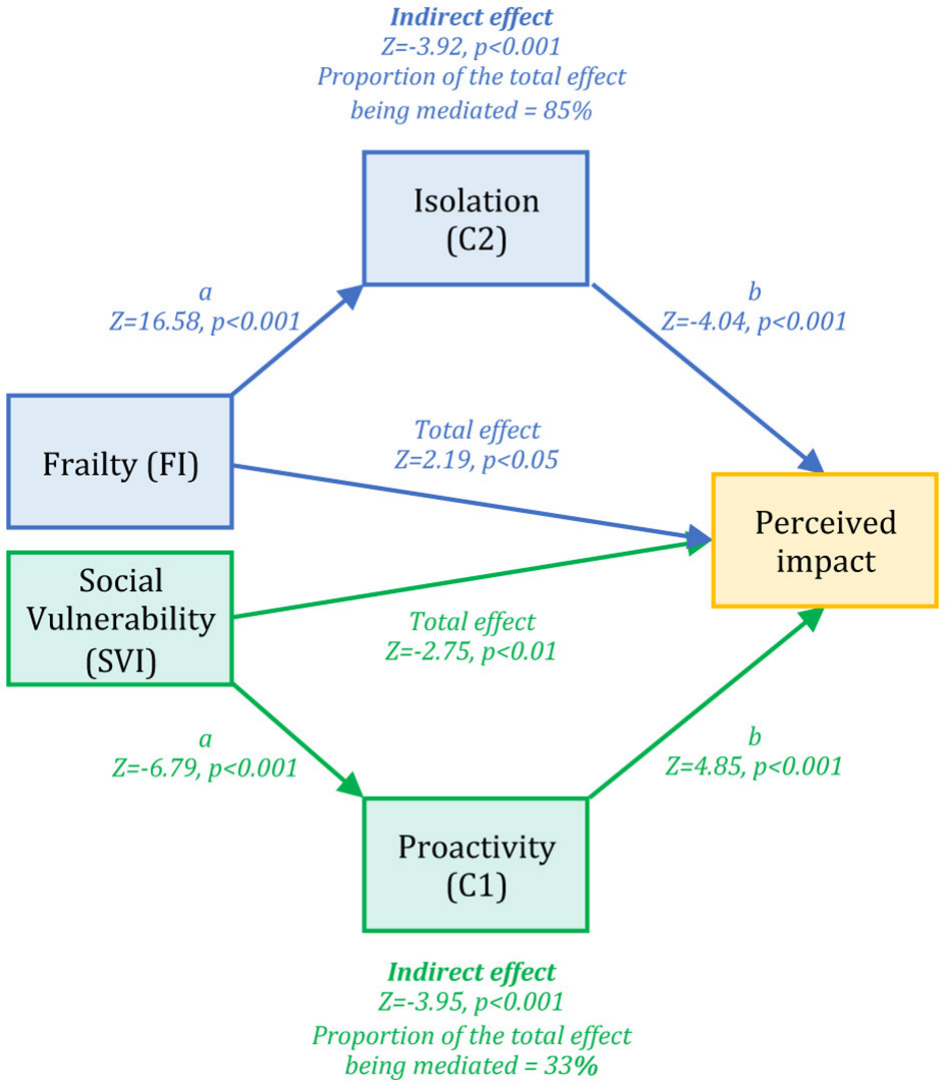
Frailty and Social Vulnerability paths. The figure displays results emerged from the path analysis, including two different paths assessed separately, both converging on a common outcome, i.e., the individual perception of the impact of Covid-19 outbreak on health (yellow box). In the Frailty path (blue color) we observed a significant mediation effect of the independent component Isolation (C2) on the relationship between the frailty index (FI) and the perceived impact of Covid-19 outbreak on health. In the Social Vulnerability path we found a significant mediation effect of the independent component Proactivity (C1) on the relationship between the social vulnerability index (SVI) and the perceived impact of Covid-19 outbreak on health.

In the *Frailty* path (Figure 2, blue color) we assessed the presence of a mediation effect of C2 (*Isolation*), which was negatively correlated to the perceived impact of Covid-19 outbreak for health (dependent variable) and positively with FI (independent variable), on the positive relationship linking FI and the perceived impact of Covid-19 outbreak for health. We found a significant indirect effect of C2 (Sobel test *p* < 0.001), which mediates ~86% of the total effect of FI on the perceived impact of Covid-19 outbreak for health. Here, direct effect and indirect effect showed opposite signs (direct effect = 3.3, *Z* = 3.7, *p* < 0.001; indirect effect = −1.5, *Z* = −3.92, *p* < 0.001), suggesting that C2 exerted a suppression effect on the relationship between frailty and the perceived impact of Covid-19 on health (31).

In the *Social Vulnerability* path (Figure 2, green color) we assessed the presence of a mediation effect of C1 (*Proactivity*), which was positively correlated with the perceived impact of Covid-19 outbreak for health (dependent variable) and negatively correlated with SVI (independent variable), on the negative relationship linking SVI and the perceived impact of Covid-19 outbreak for health. Here we observed a significant indirect effect of problem-oriented coping (Sobel test *p* < 0.001), which mediates ~33% of the total effect of SVI on perceived impact of Covid-19 outbreak for health.

## Discussion

Social distancing measures and indoor space isolation have been applied as effective actions to contain SARS-CoV-2 contagion. Though differently adopted by governments, these measures entered everyday life for millions of people in a few days. Digital solutions to communicate with others and limited physical contacts will characterize our future for a while. It is however not negligible that this may have detrimental effects on the individual psychophysical health status and the well-being of the society. Humans live in a social contextual world and need social interactions to enhance the equilibrium of mind and brain, especially in case of vulnerable individuals.

In line with previous literature, frailty showed a significant increase with age. However, as expected, social vulnerability showed a different pattern. Indeed, younger people and elders appear the most vulnerable age categories. Women and people between 35 and 64 y.o. represented the less socially vulnerable categories in our sample. This evidence may suggest that the social distancing period during the lockdown phase was critical for those people whose significant relationships are held with contacts outside the family network (e.g., friends, colleagues, or mates for young adults, as well as relatives and next of kin for elders living alone). At the same time, individuals less engaged with indoor childcare or caregiving duties (i.e., men) may have suffered the most. This finding diverged from classical studies about social vulnerability [e.g., see Andrew et al. (32)], which reported that both frailty and social vulnerability correlate each other and have a linear increase with age, with women showing higher index values than men. However, the extraordinary and unprecedented observation time of this study, together with the fact that we computed FI and SVI on a sample covering all adult ages, and not only elderly, may account for differences with these classical studies.

Both FI and SVI can be considered as predictors of the perceived impact of Covid-19 outbreak on health. However, while FI was directly related to the perceived severity of the Covid-19 impact on health—people having a higher FI perceiving the Covid-19 outbreak as more severe than individuals with lower FI—we observed a negative relationship between SVI and the perceived impact of Covid-19 outbreak for health, indicating that the subjects with higher social vulnerability were perceiving the impact of the Covid-19 outbreak as less severe than people displaying lower SVI. In particular, this latter result is confirming a previous suggestion (33). The lack of social contacts and loneliness made individuals less aware of the impact of the Covid-19 outbreak for health. Conversely, it is straightforward to understand the reason why people with high FI perceived the Covid-19 outbreak impact as more severe. Indeed, we believe that the presence of physical symptoms and/or preexistent diseases may possibly enhance the perceived feeling of being in danger during the Covid-19 pandemic, as it represents a life-threaten acute event that may alter preexistent psycho-physical integrity. Testing two possible indirect effects through which FI and SVI might relate to the perceived impact of the Covid-19 outbreak for health, we found that both frailty and social vulnerability paths showed significant mediation effects. In line with our previous work (18), the *Frailty* path revealed that the *Isolation* component (C2, including two different loneliness measures) had a suppression effect (31) on the relationship linking FI with the perceived impact of Covid-19 outbreak for health, possibly making those individuals experiencing a greater degree of loneliness less aware of the impact of the Covid-19 outbreak for health. Again, the presence of a larger social network increases the probability to have friends, relatives or colleagues who have been infected and thus to judge the impact of the outbreak as more severe. The *Social Vulnerability* path highlighted the mediation effect of *Proactivity* (C1, including variables related to empathy, social support, active and positive coping strategies) to the negative relationship linking SVI to the perceived seriousness of Covid-19 outbreak impact for health. This might indicate that, in a condition of social vulnerability and lack of connectedness, the presence of empathic skills and proactive coping strategies can reduce the detrimental effect of SVI, increasing people awareness about the health impact of Covid-19.

Finally, there are some limitations to the present work mainly related to the cross-sectional nature of the study that prevents us to generalize results and draw inference on possible changes over time. Data collection based on a convenience-based sampling and relying on self-report questionnaires may hinder the generalization of our findings to the general population. Thus, only future replication studies on larger samples and including younger (<18 y.o.) and older (>65 y.o.) individuals, can confirm the reliability of present results and overcome limitations of our study design.

## Conclusion

Our findings underline the dangers of social isolation in general population, as well as the importance of empathic skills and active coping strategies in promoting the individuals' psychosocial adaptation to a threatening event, like the Covid-19 pandemic. Frailty and social vulnerability, which contributed in explaining the individual perception of the perceived impact of Covid-19 emergency on health, were indeed influenced by proactive attitudes/behaviors and social isolation.

Measure as frailty and social vulnerability indices, coupled with information on personal psychological and emotional attitudes, may thus be helpful to monitor vulnerable populations, acting early to prevent social distancing from becoming social isolation. Social isolation in students and elderly has been linked to increased risk of mental illness as well as of cognitive decline and immune dysregulation (33–35). Moreover, it reduces resilience factors such as self-worth, sense of purpose, and feeling valued (36). These effects may lead to adverse health outcomes and increase susceptibility to infections. Health care systems and society communities must thus consider without further delay the psychosocial burden of social distancing, finding shared support strategies to keep individual engaged and motivated and screening for mental and physical symptoms.

## Data Availability Statement

The raw data supporting the conclusions of this article are available at the following doi: 10.5281/zenodo.5082071.

## Ethics Statement

The studies involving human participants were reviewed and approved by IUSS-University of Pavia Ethics Committee. The patients/participants provided their written informed consent to participate in this study.

## Author Contributions

CCe, MC, GS, and CCr conceptualized the work and designed the analysis. CCe, GS, CG, AD, and CCr performed data collection and analysis. CCe, MC, AD, SC, TV, and CCr interpreted the results. CCe and CCr wrote the main manuscript text. All authors reviewed and approved the final version of the work.

## Conflict of Interest

The authors declare that the research was conducted in the absence of any commercial or financial relationships that could be construed as a potential conflict of interest.

## Publisher's Note

All claims expressed in this article are solely those of the authors and do not necessarily represent those of their affiliated organizations, or those of the publisher, the editors and the reviewers. Any product that may be evaluated in this article, or claim that may be made by its manufacturer, is not guaranteed or endorsed by the publisher.
