# Supplementary material for: Identifying Frail Populations for Disease Risk Prediction and Intervention Planning in the Covid-19 Era: A Focus on Social Isolation and Vulnerability
Source: Front Psychiatry. 2021 Aug 20;12:626682. doi: 10.3389/fpsyt.2021.626682 (PMC8417585; doi:10.3389/fpsyt.2021.626682)
Supplement: Supplementary file 1 [file Data_Sheet_1.docx]

**Appendix A.** The 30-item Frailty Index (FI)

| 1 | Hypertension |
| --- | --- |
| 2 | Cardiovascular disorders |
| 3 | Chronic obstructive pulmonary disease |
| 4 | Gastric diseases |
| 5 | Renal failure |
| 6 | Cirrhosis |
| 7 | Diabetes |
| 8 | Thyroid disease |
| 9 | Cancer |
| 10 | Genitourinary diseases |
| 11 | Osteoporosis |
| 12 | Peripheral artery disease |
| 13 | Neurological diseases |
| 14 | Vision or hearing impairments |
| 15 | Psychiatric diseases |
| 16 | Anxiety |
| 17 | Depression |
| 18 | Stress |
| 19 | Attention deficit |
| 20 | Faintness |
| 21 | Headache |
| 22 | Dizziness |
| 23 | Diffuse pain |
| 24 | Chest pain |
| 25 | Back pain |
| 26 | Nausea |
| 27 | Soreness |
| 28 | Weakness |
| 29 | Physical inactivity |
| 30 | Mobility disability (inability to walk 400 m) |

**Appendix B.** The 30-Item Social Vulnerability Index (SVI)

| *Socio-economic status* | |
| --- | --- |
| 1 | Education (< 8 years) |
| 2 | Working status (unemployed/retired/housemaker) |
| 3 | No home ownership |
| 4 | No Smartphone/iPhone ownership |
| 5 | No PC/Tablet ownership |
| *Living situation* | |
| 6 | Marital status (single/widow/separated) |
| 7 | Lives alone |
| 8 | Lives with less than two other people |
| 9 | Lives in a rural area or a small town |
| *Leisure activities* | |
| 10 | How often go to clubs, church or community centers (< one a month) |
| 11 | How often go to gym or sport clubs (< one a month) |
| 12 | How often do charity work (< one a month) |
| *Socially oriented activities of daily living* | |
| 13 | How often look after kids (< one a month) |
| 14 | How often look after grandchildren (< one a month) |
| 15 | How often look after parents (< one a month) |
| 16 | How often look after grandparents (< one a month) |
| 17 | How often look after friends, other people (< one a month) |
| 18 | How often take care of pet (< one a month) |
| 19 | How often travel (> six months ago) |
| 20 | How often have face-to-face contacts with not relatives or friends (< one a month) |
| 21 | How often meet first-degree relatives (< one a month) |
| 22 | How often meet second-degree relatives (< one a month) |
| 23 | How often meet friends (< one a month) |
| 24 | How often talk by phone with first-degree relatives (< one a month) |
| 25 | How often talk by phone with second-degree relatives (< one a month) |
| 26 | How often talk by phone with friends (< one a month) |
| 27 | How often chat with first-degree relatives (< one a month) |
| 28 | How often chat with second-degree relatives (< one a month) |
| 29 | How often chat with friends (< one a month) |
| 30 | No social contacts in the last week |
