# Supplementary material for: Identifying Frail Populations for Disease Risk Prediction and Intervention Planning in the Covid-19 Era: A Focus on Social Isolation and Vulnerability
Source: Front Psychiatry. 2021 Aug 20;12:626682. doi: 10.3389/fpsyt.2021.626682 (PMC8417585; doi:10.3389/fpsyt.2021.626682)
Supplement: Supplementary file 2 [file Data_Sheet_2.docx]

**SUPPLEMENTARY MATERIALS**

**Identifying frail populations for disease risk prediction and intervention planning in the Covid-19 era: a focus on social isolation and vulnerability**

*Authors*

Chiara Cerami, Marco Canevelli, Gaia Chiara Santi, Caterina Galandra, Alessandra Dodich, Stefano F Cappa, Tomaso Vecchi, Chiara Crespi

**Table S1. Interpretation of principal components**

| **A: Component** | **B: Variables** | **C: Functions** |
| --- | --- | --- |
| ***#C1: Proactivity*** | IRI-EC | Emotional Empathy |
|  | IRI-PT | Cognitive Empathy |
|  | ILS-S | Social support |
|  | COPE-NVI-25 PO | Active coping strategies |
|  | COPE-NVI-25 PA | Positive coping strategies |
|  | COPE-NVI-25 SS | Social support coping strategies |
| ***#C2: Isolation*** | ILS-G | Loneliness |
|  | ILS-E | Loneliness |
| ***#C3: Inactivity*** | TAS-20 | Alexithymia |
|  | COPE-NVI-25 TO | Transcendent coping style |
|  | COPE-NVI-25 AS | Avoidance coping style |

The table illustrates the three principal components – Proactivity, Isolation, Inactivity – obtained as a result of the data reduction procedure performed on psycho-socio-emotional variables, including different facets of loneliness, empathy, coping styles and alexithymia (see main text, paragraph 3.3). In particular, for each principal component (Column A) we listed the variables included (Column B) and the corresponding function (Column C).

*Table legend: C1=component 1; C2=component 2; C3=component 3; IRI-EC=Interpersonal Reactivity Index, Empathic concern subscale; IRI-PT= Interpersonal Reactivity Index, Perspective taking subscale; ILS-S=Italian Loneliness Scale, Social support subscale; COPE-NVI-25 OP=Coping Orientation to Problem Experienced-New Italian Version, Problem orientation subscale; COPE-NVI-25 PA=Coping Orientation to Problem Experienced-New Italian Version, Positive attitude subscale; COPE-NVI-25 SS=Coping Orientation to Problem Experienced-New Italian Version, Social Support subscale; ILS-G=Italian Loneliness Scale, General Loneliness subscale; ILS-E=Italian Loneliness Scale, Emotional Loneliness subscale; TAS-20=Toronto Alexithymia Scale; COPE-NVI-25 OP TO=Coping Orientation to Problem Experienced-New Italian Version, Transcendent orientation subscale; COPE-NVI-25 AS=Coping Orientation to Problem Experienced-New Italian Version, Avoidance strategies subscale.*

**Table S2. Correlation results**

|  | *Perceived Covid-19 Impact on Health* | *FI* | *SVI* | *C1* | *C2* | *C3* |
| --- | --- | --- | --- | --- | --- | --- |
| *Perceived Covid-19*  *Impact on Health* | - | r=0.062  **p=0.028** | r=-0.077  **p=0.006** | r=0.147  **p=0.000** | r=-0.076  **p=0.007** | r=0.007  p=0.804 |
| *FI* |  | - | r=-0.004  p=0.888 | r=-0.003  p=0.918 | r=0.424  **p=0.000** | r=0.182  **p=0.000** |
| *SVI* |  |  | - | r=-0.188  **p=0.000** | r=0.044  p=0.119 | r=0.013  p=0.657 |
| *C1* |  |  |  | - | r=0.000  p=1.000 | r=0.000  p=1.000 |
| *C2* |  |  |  |  | - | r=0.000  p=1.000 |
| *C3* |  |  |  |  |  | - |

The table reports the correlation results taking into account the perceived impact of Covid-19 on health, the frailty (FI) and the social vulnerability (SVI) indices, and the loading factors of the three principal components resulted from the PCA (C1, C2, C3). *Table legend: FI=frailty index; SVI=social vulnerability index; C1=component 1, Proactivity; C2=component 2, Isolation; C3=component 3, Inactivity. Bold values indicate statistical significance at the p<0.05 level.*
